# Hospital A.C.Camargo

**DOI:** 10.1186/1753-6561-7-S2-A3

**Published:** 2013-04-04

**Authors:** Fernando Augusto Soares

**Affiliations:** 1 Hospital A.C.Camargo, São Paulo, Brazil

## 

Private non-profit making institution created in 1953, Hospital A.C.Camargo is pioneer and one of the world’s biggest cancer treatment, teaching and research center. In an integrated and multidisciplinary manner, it operates in the prevention, diagnosis and ambulatory and surgical treatment of over 800 types of cancer identified by Medicine, divided into more than 40 specialties.

Every year its team treats about 15 thousand new patients from different corners of the country and abroad, totaling over one million procedures (consultations, lab and imaging tests, hospitalizations, surgeries, chemotherapy and radiotherapy, among others).

The A.C.Camargo Hospital Complex has over 62 thousand m² of built area, having one of the largest structures in the world of hospitalization in Oncology, with a total of 440 beds. It also has 62 chemotherapy rooms, 22 surgical rooms and the most complete technological assets in oncology in Latin America.

Its clinical body consists of a closed team of over 500 specialists, majority with masters and doctorate degrees. The dedication and interaction of these professionals in the interdisciplinary activities results in a treatment with better success rates, only comparable to those of the largest oncology centers in the world.

In 2012, it achieved the International Certification by the Canadian Council for Health Services Accreditation (CCHSA), certifying the efficiency of its processes in relation to patient safety.

In the teaching area, A.C.Camargo created the first Residency in Oncology in the country, in 1953, graduating its one thousandth resident in 2010. It is also responsible for the training of one in every three active oncologists in Brazil. The graduation created in 1997 is the only one in a private hospital that is accredited by the Ministry of Education and that obtained the highest score by CAPES during all this decade , thus becoming the best Oncology training center in the country among public and private schools and one of the two best in Medicine.

In the researching area, there is the biggest research structure in Latin America, the CIPE – International Research Center. With 4 thousand square meters, the structure of CIPE A.C.Camargo consists of research groups that work in labs with state of the art equipments and that, integrated to the entire clinical body of the hospital, conduct leading scientific projects. In 2000 it centralized the Cancer Genome in Brazil funded by FAPESP and Ludwig Institute.

It has the largest Tumor Bank and tumor tissue collection in Latin America, with 30,000 samples and the biggest scientific production of the area, with over a thousand papers published in the past decade in the major high-impact international magazines. In 2010 it was responsible for 87% of the scientific oncology production in the country.

